# Atorvastatin treatment modulates the interaction between leptin and adiponectin, and the clinical parameters in patients with type II diabetes

**DOI:** 10.3892/etm.2013.1347

**Published:** 2013-10-15

**Authors:** SAYER I. AL-AZZAM, ASEM M. ALKHATEEB, KAREM H. ALZOUBI, RAYA N. ALZAYADEEN, MERA A. ABABNEH, OMAR F. KHABOUR

**Affiliations:** 1 Department of Clinical Pharmacy, Faculty of Pharmacy, Jordan University of Science and Technology, Irbid 22110, Jordan; 2 Department of Biotechnology and Genetic Engineering, Faculty of Science and Art, Jordan University of Science and Technology, Irbid 22110, Jordan; 3 Department of Medical Laboratory Sciences, Faculty of Applied Medical Sciences, Jordan University of Science and Technology, Irbid 22110, Jordan

**Keywords:** adiponectin, leptin, statins, insulin resistance, lipid panel

## Abstract

The aim of this study was to examine the effect of atorvastatin treatment on levels of leptin, adiponectin and insulin resistance, and their correlation with clinical parameters, in patients with type II diabetes. Patients with diabetes (n=394) were divided into two groups, comprising 161 patients who received 20 mg/day atorvastatin (statin group), and 233 patients who did not receive statins (statin-free group). The results showed that atorvastatin treatment of patients with diabetes was not associated with changes in leptin, adiponectin, the leptin/adiponectin (L/A) ratio or homeostasis model assessment-insulin resistance (HOMA-IR). However, low-density lipoprotein cholesterol (LDL-C), triglycerides (TG) and total cholesterol (Tchol) were positively correlated with leptin and L/A ratio in the statin group only (P<0.05). By contrast, high-density lipoprotein cholesterol (HDL-C) showed a significant positive correlation with adiponectin in the statin and statin-free groups (P<0.05). Additionally, a positive correlation was found between HOMA-IR and glycated hemoglobin (HbA1c), and TG, in both groups, whereas Tchol was positively correlated with HOMA-IR in the statin group only (P<0.05). When multivariate analysis was performed with HOMA-IR as the dependent variable, and with adjustment for age, body mass index (BMI) and waist circumference, HbA1c was found to be a significant predictor of HOMA-IR or insulin resistance. In conclusion, atorvastatin treatment may have several effects on the interaction between leptin and adiponectin, and on clinical parameters in patients with type II diabetes.

## Introduction

Atorvastatin, which belongs to the statin family of drugs, is used in the treatment of hyperlipidemia, which is commonly associated with type II diabetes (1). The drug is known to achieve a significant reduction in low-density lipoprotein cholesterol (LDL-C), with a moderate reduction in triglycerides (TG), via the inhibition of the 3-hydroxy-3-methylglutaryl-CoA (HMG-CoA) reductase enzyme (2). Atorvastatin use may also result in the primary prevention of cardiovascular disease in patients with type II diabetes (3). In addition, it is known for having extra-lipid (pleiotropic) effects, such as the modulation of circulating leptin and adiponectin levels (4–7).

Leptin and adiponectin are two major adipokines that have been implicated in metabolic homeostasis (8–11). Leptin is primarily produced in adipose tissue and is important in the regulation of body weight (12). By contrast, adiponectin is known for its antidiabetes and antiatherosclerosis properties, which are significantly reduced in metabolic disorders, particularly in hyperlipidemia (13,14). In animal models, rabbits treated with atorvastatin showed decreased levels of circulating leptin (15). In humans, atorvastatin lowered circulating leptin in patients with type II diabetes (16) and increased adiponectin levels in individuals with a high cardiovascular risk (17). Therefore, circulating leptin and adiponectin levels may be affected by atorvastatin treatment. In this study, the effect of atorvastatin treatment on the interaction between leptin and adiponectin, and on clinical parameters was examined in patients with type II diabetes.

## Patients and methods

### Study design and sampling

This cross-sectional study was approved by the Institutional Review Boards (IRB) of the Jordan University of Science and Technology (Irbid, Jordan) and the Ministry of Health (Amman, Jordan). All patients signed a consent form prior to participation. Recruitment was conducted between October 2009 and June 2010. Recruitment took place at the Medical Health Center of Jordan University of Science and Technology, King Abdullah University Hospital (Irbid, Jordan) and Princess Basma Teaching Hospital (Irbid, Jordan).

### Subject description

A total of 394 patients with type II diabetes, aged ≥18 years, participated in the study. Exclusion criteria were: The presence of liver disease, the elevation of transaminase or creatine kinase (CK) levels to >1.5-fold the upper normal limit at baseline, the presence of atrioventricular block and/or sinus bradycardia, the presence of acute or chronic renal failure (as per specialist physician diagnosis), evidence of electrolyte disturbances, cases of acute cerebrovascular disease or myocardial infarction within the preceding three months, evidence of alcohol abuse, patients with hypothyroidism, evidence of myopathy, pregnant patients, females of premenopausal age and patients who had any change in medication during the two months preceding their participation.

### Data collection

During the clinic visit, the study procedure and goals were explained to patients verbally and through a consent form. Patients who were approved for participation were interviewed by a trained researcher using a structured questionnaire. Information regarding clinical history and current drug regimen was obtained from medical files. In addition, patient variables, such as height, weight, blood pressure and waist circumference, were measured during the clinic visit.

### Blood sampling and handling

Overnight-fasting blood samples were drawn from participants who fit the study criteria by a specialized laboratory technician. Each sample was distributed in an evacuated EDTA tube (5 ml blood) and an anticoagulant-free plain tube (10 ml blood). Blood samples in plain tubes were centrifuged at 4,000 × g for 4 min. Part of the serum sample was used for biochemical measurements. The remaining serum was distributed in small aliquots (100 μl), which were later used for enzyme-linked immunosorbent assay (ELISA) of leptin and adiponectin. Whole blood samples of EDTA tubes were used for glycated hemoglobin (HbA1c) measurement.

### Body mass index (BMI)

Using the reported height and weight of the patients, BMI was calculated using the following equation: BMI = weight (kg)/[height (m)]^2^(18,19).

### Index of insulin resistance

The degree of insulin resistance was evaluated using the homeostatic model assessment devised by Matthews *et al*(20), and calculated using the following formula: Homeostasis model assessment-insulin resistance (HOMA-IR) = fasting serum insulin (μU/ml) × fasting plasma glucose (mmol/l)/22.5 (21).

### Biochemical assays

The levels of fasting plasma glucose (FPG), HbA1c, total cholesterol (Tchol), LDL-C, high-density lipoprotein cholesterol (HDL-C), TG, aspartate aminotransferase (ALT), alanine aminotransferase (AST), serum creatinine (SrCr) and CK were measured in serum at the laboratories of King Abdullah University Hospital using a Roche automated clinical analyzer system (Roche Diagnostics, Mannheim, Germany).

### Serum leptin and adiponectin

Serum leptin and adiponectin concentrations were measured using ELISA according to the manufacturer’s instructions (DuoSet ELISA; R&D Systems, Minneapolis, MN, USA). Absorbance was measured spectrophotometrically at 450 nm using an ELx800 Microplate Reader (BioTek Instruments, Winooski, VT, USA). The L/A ratio was calculated for each patient by dividing the serum concentration of leptin (ng/ml) by the serum concentration of adiponectin (μg/ml).

### Statistical analysis

Statistical analyses were conducted using JMP software (version 8.0; SAS Institute, Cary, NC, USA). Descriptive statistics, including the mean ± SD, were calculated for normally distributed data. The median and interquartile ranges were calculated for skewed data. The statistical significance was estimated using an unpaired Student’s t-test or two independent nonparametric tests (Wilcoxon Rank-Sum test). Bivariate correlation analysis for insulin resistance (HOMA-IR) and other parameters was performed using Spearman’s correlation. Multiple linear regression models were used to examine the determinant of HOMA-IR following logarithmic transformation. P<0.05 was considered to indicate a statistically significant difference.

## Results

### Patient characteristics

The study subjects were patients with type II diabetes, with an age range of 24–85 years (mean, 55.9±6.93 years). The patients were divided into statin and statin-free groups. The statin group included 161 patients who received 20 mg/day atorvastatin for management of hyperlipidemia. None of these patients experienced vascular events. The statin-free group, which included 233 patients, did not receive any drug belonging to the statin family. No significant differences were observed between the two groups with regard to age, BMI, blood pressure, FPG, HbA1c, insulin blood level, HOMA-IR, leptin, adiponectin, L/A ratio and TG (Table I). However, LDL-C, Tchol and waist circumference were different between the two groups (P<0.05).

### L/A ratio as a marker of insulin resistance

No significant correlation was found between HOMA-IR and the L/A ratio in either the statin or the statin-free groups (Table II). Additionally, no correlation was observed between leptin or adiponectin levels and HOMA-IR.

### Effect of the use of atorvastatin on the correlation between leptin, adiponectin and HOMA-IR, and clinical parameters in patients with type II diabetes

Correlations were investigated between atorvastatin treatment, leptin and adiponectin levels and clinical parameters in the patients (Table III). In the statin group, LDL-C, TG and Tchol showed a significant positive correlation with leptin and the L/A ratio. No such correlation was observed in the statin-free group. Conversely, HDL-C showed a significant positive correlation with adiponectin in the statin and statin-free groups. TG was negatively correlated with adiponectin in the statin-free, but not in the statin, group (Table III).

HOMA-IR was positively correlated with HbA1c and TG in both groups. Tchol showed a significant positive correlation with HOMA-IR in the statin group, whereas HDL-C showed a significant negative correlation with HOMA-IR in the statin-free group (Table III).

When multivariate linear regression analysis was conducted with HOMA-IR as the dependent variable, HbA1c and HDL-C were significant predictors of HOMA-IR. However, following adjusting for age, BMI and waist circumference, HbA1c remained as a significant predictor of HOMA-IR (Table IV).

## Discussion

In this study, it was shown that, under atorvastatin treatment, leptin levels and L/A ratio were correlated with the lipid profile measures, LDL-C, TG and Tchol levels. Additionally, atorvastatin treatment influenced the correlation between insulin resistance and Tchol and HDL-C levels, but not the correlation between glycemic control and TG, among patients with type II diabetes.

Statins are known for their pleiotropic effects (4,5). However, reports are conflicting as to whether statins have a potential insulin-sensitizing effect (22–25). The data from the present study indicate that the use of atorvastatin did not have a significant effect on an insulin resistance measure (HOMA-IR).

The effects of statins on serum leptin and adiponectin levels are controversial (15,26,27). It has been reported that simvastatin significantly increased insulin and leptin levels in patients with hypercholesterolemia (27). However, in hypercholesterolemic rabbits, atorvastatin was observed to reduce leptin levels following six weeks of therapy (15). In one study that examined patients with coronary heart disease, simvastatin treatment resulted in a significant decrease in blood leptin (26). With regard to adiponectin, it has been reported that simvastatin had no effect on adiponectin blood levels in hypercholesterolemic, hypertensive subjects following two months of treatment (28). Similarly, atorvastatin did not alter adiponectin concentrations in subjects with combined hyperlipidemia (29). However, other studies have reported that atorvastatin increased adiponectin blood levels in subjects with hyperlipidemia (30) or cardiovascular risk factors (17). The results of the present study are consistent with the observation that atorvastatin does not significantly modify leptin and adiponectin blood concentrations.

In this study, adiponectin levels were positively correlated with HDL-C, whereas leptin and the L/A ratio were positively correlated with LDL-C, TG and Tchol levels in patients receiving atorvastatin. These results are consistent with cumulative evidence reporting increased leptin levels and decreased adiponectin levels in patients with metabolic syndrome (10,31–33).

Atorvastatin treatment did not influence the correlation between HbA1c and TG levels, and insulin resistance. However, in the statin group, a positive correlation was found between insulin resistance and Tchol levels. These results are consistent with previous studies showing a negative correlation between a generally more favorable lipid profile and insulin resistance (34,35).

The results of the present study do not appear to be associated with the prevention of vascular events in patients with type II diabetes mellitus treated with atorvastatin (3), as this group of patients was not represented in this study. New onset diabetes (NOD) associated with statin use represents an emerging issue (36–37). A recent study showed that different types and doses of statins exhibit different potential for causing NOD (38). For instance, pravastatin (40 mg/day), rosuvastatin (20 mg/day) or atorvastatin (80 mg/day) were observed to be associated with NOD compared with a placebo-control (38). Moreover, the association of statins with NOD appears to be dose-dependent (39,40). For example, atorvastatin at doses of 10–40 mg/day showed a significantly lower incidence for NOD compared with an 80 mg/day dose in patients with between two and four NOD risk factors (39). In the present study, only patients on a 20 mg/day dose of atorvastatin were enrolled. Thus, the present results are less likely to be associated with NOD.

In conclusion, atorvastatin appears to exert multiple effects on the interaction between leptin, adiponectin and the L/A ratio, and clinical parameters, in patients with type II diabetes.

## Figures and Tables

**Table I:** Body and biochemical parameters of the statin and statin-free groups.

Parameter	Statin-free group^a^ (n=233)	Statin group^a^ (n=161)	P-value
Gender (F/M, n)	132/101	81/80	0.21
Smoking status [n (%)]	43 (18.45)	33 (20.50)	0.14
Age (years)	55.24±10.23	56.74±8.75	0.13
BMI (kg/m^2^)	31.38±5.56	31.07±5.45	0.58
Waist circumference (cm)	100.28±16.11	105.31±16.86	<0.01
SBP (mmHg)	131.36±17.76	131.70±16.74	0.86
DBP (mmHg)	83.04±13.45	83.44±11.61	0.78
HbA1c (%)	7.42±1.67	7.34±1.78	0.65
LDL-C (mmol/l)	3.31±1.07	2.92±1.12	<0.01
FPG (mmol/l)	9.70±5.86	9.31±3.92	0.44
Tchol (mmol/l)	5.23±1.20	4.78±1.50	<0.01
Adiponectin (μg/ml)	2.10±0.84	2.06±0.77	0.64
HDL-C (mmol/l)	1.1 (0.91–1.33)	1.1 (0.92–1.20)	0.59
TG (mmol/l)	1.88 (1.3–2.75)	1.85 (1.33–2.71)	0.79
Insulin (μU/ml)	9.61 (6.86–16.45)	10.62 (6.92–16.67)	0.41
Leptin (ng/ml)	1.68 (0.98–2.60)	1.69 (1.04–3.05)	0.77
L/A	0.84 (0.47–1.48)	0.89 (0.41–1.56)	0.69
HOMA-IR	4.04 (2.28–7.20)	4.29 (2.33–6.50)	0.68

a With the exception of gender and smoking status, values are presented as the mean ± SD or median (interquartile range, IQR).

F, female; M, male; BMI, body mass index; SBP, systolic blood pressure; DBP, diastolic blood pressure; HbA1c, glycated hemoglobin; LDL-C, low-density lipoprotein cholesterol; FPG, fasting plasma glucose; Tchol, total cholesterol; HDL-C, high-density lipoprotein cholesterol; TG, triglycerides; L/A, leptin-to-adiponectin ratio; HOMA-IR, homeostasis model assessment-insulin resistance.

**Table II:** Spearman’s correlation for serum leptin, adiponectin and L/A with HOMA-IR.

		HOMA-IR	
		
Parameter	Atorvastatin	ϱ^a^	P-value
L/A	Yes	0.0948	0.1492
	No	0.1087	0.1725
Leptin	Yes	0.0231	0.7725
	No	0.1070	0.1031
Adiponectin	Yes	−0.1488	0.0612
	No	−0.0163	0.8050

a Spearman’s correlation coefficient. L/A, leptin-to-adiponectin ratio; HOMA-IR, homeostasis model assessment-insulin resistance rate.

**Table III:** Spearman’s correlation for serum leptin, adiponectin, L/A and HOMA-IR with various parameters in the atorvastatin (statin) and statin-free groups.

		Leptin		Adiponectin		L/A		HOMA-IR	
					
Parameter	Atorvastatin	ϱ	P-value	ϱ	P-value	ϱ	P-value	ϱ	P-value
HbA1c	Yes	−0.0208	0.7937	−0.1065	0.1802	0.0076	0.9243	0.2900	0.0002
	No	0.0282	0.6695	−0.0002	0.9981	−0.0041	0.9512	0.2864	<0.0001
HDL-C	Yes	0.0189	0.8123	0.2124	0.0068	−0.1271	0.1080	−0.1502	0.0588
	No	0.0598	0.3637	0.1509	0.0212	−0.0046	0.9441	−0.1751	0.0074
LDL-C	Yes	0.2002	0.0131	−0.0184	0.8217	0.1858	0.0215	0.1054	0.1976
	No	0.0298	0.6340	0.0466	0.4827	0.0117	0.8597	−0.0859	0.1954
TG	Yes	0.2105	0.0074	−0.1036	0.0987	0.2683	0.0006	0.3412	<0.0001
	No	0.0754	0.2517	−0.1553	0.0176	0.1191	0.0696	0.1850	0.0046
Tchol	Yes	0.1994	0.0112	−0.0646	0.4154	0.2108	0.0073	0.2611	0.0009
	No	0.0077	0.9064	0.0585	0.3743	−0.0250	0.7039	−0.0001	0.9884

L/A, leptin-to-adiponectin ratio; HOMA-IR, homeostasis model assessment-insulin resistance rate; HbA1c, glycated hemoglobin; ϱ, Spearman’s correlation coefficient; HDL-C, high-density lipoprotein cholesterol; LDL-C, low-density lipoprotein cholesterol; TG, triglycerides; Tchol, total cholesterol.

**Table IV:** Multivariate linear regression analysis with HOMA-IR as the dependent variable^a^.

	Multivariate analysis		Multivariate analysis (adjusted^b^)	
		
Parameter	β	P-value	β	P-value
HbA1c	0.112	<0.0001	0.1180	<0.0001
Atorvastatin	−0.022	0.5992	−0.0230	0.7534
HDL-C	−0.529	0.001	−0.5290	0.0009
LDL-C	−0.173	0.0767	−0.1890	0.5035
TG	−0.026	0.5831	−0.0270	0.5688
Tchol	0.178	0.0746	0.1792	0.0174

a HOMA-IR was log transformed;

b adjusted for age, BMI and waist circumference. β, regression coefficient by multiple linear regression analysis.

HOMA-IR, homeostasis model assessment-insulin resistance; HbA1c, glycated hemoglobin; HDL-C, high-density lipoprotein cholesterol; LDL-C, low-density lipoprotein cholesterol; TG, triglycerides; Tchol; total cholesterol.
